# The Efficacy of Umbelliferone, Arbutin, and N-Acetylcysteine to Prevent Microbial Colonization and Biofilm Development on Urinary Catheter Surface: Results from a Preliminary Study

**DOI:** 10.1155/2016/1590952

**Published:** 2016-04-05

**Authors:** Tommaso Cai, Luca Gallelli, Francesca Meacci, Anna Brugnolli, Letizia Prosperi, Stefani Roberta, Cristina Eccher, Sandra Mazzoli, Paolo Lanzafame, Patrizio Caciagli, Gianni Malossini, Riccardo Bartoletti

**Affiliations:** ^1^Department of Urology, Santa Chiara Regional Hospital, 38123 Trento, Italy; ^2^Department of Health Science, School of Medicine, University of Catanzaro, 88100 Catanzaro, Italy; ^3^Clinical Pharmacology and Pharmacovigilance Unit, Mater Domini University Hospital, 88100 Catanzaro, Italy; ^4^Sexually Transmitted Diseases Center, Santa Maria Annunziata Hospital, 50012 Florence, Italy; ^5^Department of Public Health, University of Verona, 37100 Verona, Italy; ^6^Department of Microbiology, Santa Chiara Regional Hospital, 38123 Trento, Italy; ^7^Department of Laboratory Medicine, Santa Chiara Regional Hospital, 38123 Trento, Italy; ^8^Department of Urology, University of Florence, 50100 Florence, Italy

## Abstract

We evaluated, in a preliminary study, the efficacy of umbelliferone, arbutin, and N-acetylcysteine to inhibit biofilm formation on urinary catheter. We used 20 urinary catheters: 5 catheters were incubated with* Enterococcus faecalis* (control group); 5 catheters were incubated with* E. faecalis* in presence of umbelliferone (150 mg), arbutin (60 mg), and N-acetylcysteine (150 mg) (group 1); 5 catheters were incubated with* E. faecalis* in presence of umbelliferone (150 mg), arbutin (60 mg), and N-acetylcysteine (400 mg) (group 2); and 5 catheters were incubated with* E. faecalis* in presence of umbelliferone (300 mg), arbutin (60 mg), and N-acetylcysteine (150 mg) (group 3). After 72 hours, planktonic microbial growth and microorganisms on catheter surface were assessed. In the control group, we found a planktonic load of ≥10^5^ CFU/mL in the inoculation medium and retrieved 3.69 × 10^6^ CFU/cm from the sessile cells adherent to the catheter surface. A significantly lower amount in planktonic (*p* < 0.001) and sessile (*p* = 0.004) bacterial load was found in group 3, showing <100 CFU/mL and 0.12 × 10^6^ CFU/cm in the incubation medium and on the catheter surface, respectively. In groups 1 and 2, 1.67 × 10^6^ CFU/cm and 1.77 × 10^6^ CFU/cm were found on catheter surface. Our results document that umbelliferone, arbutin, and N-acetylcysteine are able to reduce* E. faecalis* biofilm development on the surface of urinary catheters.

## 1. Introduction

In Europe, the incidence of nosocomial urinary tract infections (UTIs) associated with the presence of indwelling urinary catheters (CAUTIs) accounts for 3.55 per 1000 hospitalised patient-days with an important impact on public health costs [1]. Moreover, the presence of indwelling urinary catheter is a risk factor for development of symptomatic urinary tract infections. In fact, from 10% to 30% of patients with indwelling urinary catheters develop symptomatic UTIs [2]. Recently, the research's attention is focused on the role of bacterial biofilms in the development of CAUTIs and UTIs [3]. Biofilm formation plays an important role in the field of urology, due to its development on the surface of indwelling urinary catheters and ureteral stents and the subsequent infection, often with antibiotic resistance and development [4]. The microbial biofilm capacity to adhere on urinary catheter is due to its feature: a structured community of microorganism, with an extensive exopolymer matrix (slime) protecting the microbial cells against unfavourable environmental factors [5, 6]. Several methods have been purposed to prevent biofilm formation in the urinary tract, that is, antibiotic-impregnated catheters, silver hydrophilic coating, heparin coating, and triclosan [6], since these devices seem to not be able to improve clinical symptoms or to decrease the antibiotic use. Plants extracts are often used in urology to treat the UTIs, particularly the recurrent forms [7]. The idea is to prevent the adhesion of bacteria on urinary tract mucosa, in particular the bladder mucosa, and to avoid the biofilm formation, which are the key of recurrent UTIs development [8]. Recently, Cai and coworkers demonstrated that the use of plants extracts resulted in a significant reduction of microbial colonization in patients with indwelling urinary catheters [4]. In this study, we focused our attention on umbelliferone (7-hydroxycoumarin), arbutin, and N-acetylcysteine. In our study, umbelliferone was isolated from* Herniaria hirsuta* (Caryophyllaceae), a flowering plant, native to Eurasia and North Africa, used in folk medicine as a diuretic and to treat kidney stones [9]. Several authors showed that the extract of* Herniaria hirsuta* may contain substances that inhibit calcium oxalate crystal aggregation and might be beneficial in preventing kidney stone formation [10]. The idea to use umbelliferone in this study is based on the hypothesis that it is able to reduce the biofilm formation by preventing the biofilm “core” enucleation. On the other hand, arbutin, the active principle of bearberry, inhibits the bacterial growth by the effect of hydroquinone [11]. Finally, several studies showed that N-acetylcysteine (NAC) is able to decrease biofilm formation by reducing the production of extracellular polysaccharide matrix and promoting the disruption of mature biofilm [12]. Based on these previous evidences, this preliminary was aimed at evaluating* in vitro* efficacy of different mixtures containing umbelliferone, arbutin, and N-acetylcysteine to decrease microbial colonization and biofilm development.

## 2. Materials and Methods

### 2.1. Study Schedule

The study took place between January and March 2014 using a modified method described by Kuhn et al. [13] and Mazzoli [14]. In the first month, we tested this method to improve our skill in this analysis. The following two months were devoted to biofilm development, data collection, and results analysis.

### 2.2. Bacterial Strain

A single wild strain of* Enterococcus faecalis* (previously isolated from a patient with UTI as described by Mazzoli [14]) was used during the whole study. The strain was identified and characterized biochemically using the species identification cards of the VITEK II Semiautomated System for Microbiology (bioMérieux; BD, Italy). Antibiotic sensitivity was assayed on different antibiotic cards of the VITEK II (bioMérieux; BD, Italy) and minimum inhibitory concentration and multidrug resistant break points recommended by Clinical and Laboratory Standards Institute were used [15]. The* Enterococcus faecalis* strain, used in this experiment, showed resistance to the following antimicrobial agents: ciprofloxacin, cotrimoxazole, levofloxacin, and sulfamethoxazole.

### 2.3. Catheters and “*In Vitro*” Model

**Figure 1:**
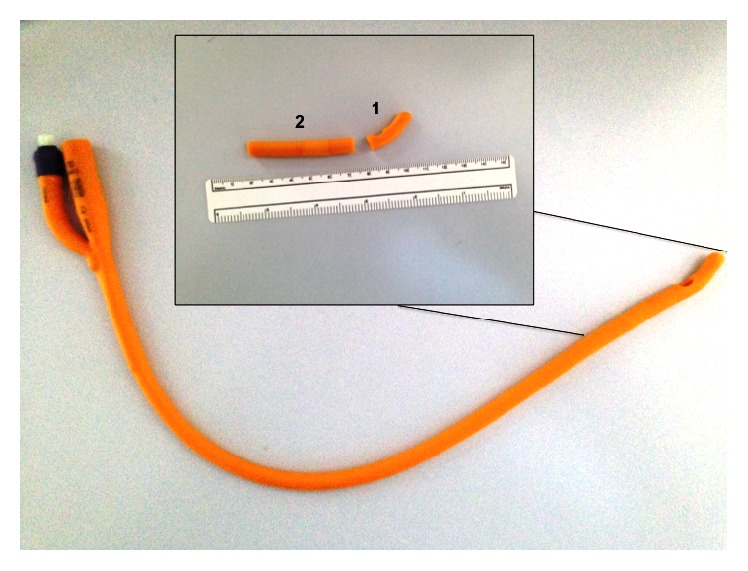
The urinary catheter preparation procedure for the experiment.

Twenty-five urinary catheters (Silkolatex® Rüsch Gold® Size Ch. 22) were used. Five catheters were used for the training phase and 20 for the experimental study. The proximal 5 cm long segment of each catheter was aseptically separated with a sterile scalpel (Figure 1). Separated bioreactors were set up for each catheter segment during the experimental phase according to the following procedure. The catheter segment was put in a Falcon tube (Falcon® Brand Products; Life Sciences Brands) and 40 mL of* E. faecalis* suspension (5 × 10^5^  colony-forming units (CFU)/mL) in Mueller-Hinton broth (Sigma-Aldrich Co., LLC., USA) was added. Then, 5 mL of different solutions was poured in each bioreactor, according to the following experimental groups:

 Control group: 0.9% sterile saline.
 Group  1: aqueous solution containing umbelliferone (150 mg), arbutin (60 mg), and N-acetylcysteine (150 mg).
 Group  2: aqueous solution containing umbelliferone (150 mg), arbutin (60 mg), and N-acetylcysteine (400 mg).
 Group  3: aqueous solution containing umbelliferone (300 mg), arbutin (60 mg), and N-acetylcysteine (150 mg).

**Figure 2:**
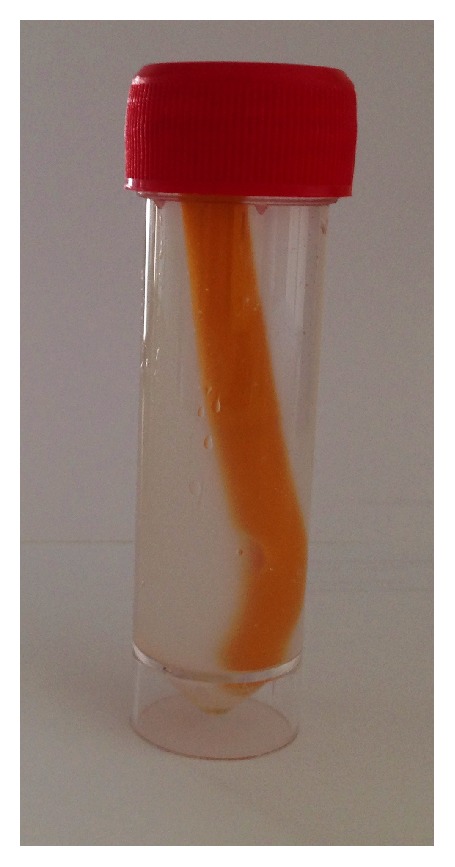
The incubation period of each catheter segment into a 50 mL Falcon tube. 45 mL is the total volume of the solution in each bioreactor.

Bioreactors were incubated at 37°C for 72 h (Figure 2).

### 2.4. Cultural Quantitative Methods

After incubation, the amount of planktonic microbial cells in the incubation medium was evaluated by plating aliquots on solid growth medium. CFU were enumerated after 24 h and expressed as CFU/mL of incubation medium, in agreement with previous paper [14]. The remaining incubation medium was discarded and each catheter segment was used for the quantification of sessile bacteria as previously described [4]. Briefly, after gentle washing in sterile saline, all collected catheter segments were placed in 5 mL of 0.9% sterile saline solution and sonicated for 15 min to release bacterial cells from the biofilm adhering to the catheter surface [2]. The resulting suspension was quantitatively cultured on solid medium to quantify the amount of* E. faecalis* cells in the biofilm. Results were presented as CFU/cm of catheter. The main outcome measure was the amount of CFU/cm, as an indicator of the quantity of biofilm formed on the catheter surface at the end of the incubation period. The rationale behind the choice of* E. faecalis* for this experiment is two main points: first,* E. faecalis* is one of the most common causes of nosocomial infections and an important etiological agent of CAUTIs [16]; second,* E. faecalis* is frequently a strong biofilm producer, strongly adhering to the surface of the urinary catheter [14] establishing persistent UTI [16].

### 2.5. Microbiological Analysis and Biofilm Evaluation

A 5 cm long segment from the tip of the catheter was used for microbiological analysis and biofilm evaluation. Microbiological analysis was performed on both inoculation broth and catheter surface. Microbiological evaluation was performed in agreement with previous paper and as described above [14]. Quantitative assessment of slime production was performed using crystal violet binding assay method according to the Christensen microwell assay [17]. Optical density (OD) measurements, read by a microreader apparatus (ETI-System S800, DiaSorin, Italy) at 630 nm with the photometer switched to the single-wave length mode, were performed in triplicate and averaged.

### 2.6. Composition and Characterization of the Extracts Used

The N-acetyl-L-cysteine used in this experiment has been obtained from Giusto Faravelli SpA, Milan, Italy. We used the* Agropyron repens* extract, 20% arbutin (HPLC), produced in China and imported from L.C.M. SpA, Milan, Italy. Moreover, the* Herniaria hirsuta* extract was produced in North Europe and imported from Solimè S.r.l., Reggio Emilia, Italy.

### 2.7. Statistical Analysis

Median and interquartile ranges (IQRs) for quantitative cultural data were computed. The amount of planktonic (CFU/mL) and sessile (CFU/cm) bacteria was compared among the experimental groups by using a Kruskal-Wallis test. Moreover, Wilcoxon rank-sum tests were used for pairwise comparisons and the levels of post hoc pairwise comparisons were adjusted using a sequential Bonferroni adjustment. The threshold for statistical significance was set at *p* < 0.05. SPSS 11.0 software (SPSS Inc., Chicago, USA) was used for the statistical analyses. We defined this study as exploratory; therefore we did not determine a power calculation.

## 3. Results

### 3.1. Training Phase

The results of the training and experimental phases are displayed below. Using 5 catheters, we documented a significant development of* Enterococcus faecalis* biofilm both in inoculation medium and on catheter surface. In the medium we documented ≥10^5^ CFU/mL development of the strain, while on the catheter surface we found 3.69 × 10^6^ median CFU per 1 cm catheter segment (IQRs: 2.28).

### 3.2. Experiment Phase

The 5 catheter segments of the control group showed ≥10^5^ CFU/mL in the incubation medium development of the strain while on the catheter surface we found 3.69 × 10^6^ median CFU per 1 cm catheter segment (IQRs: 2.28). Results of the cultural analysis at the start and at the end of the incubation period are summarized in Table 1. Initial planktonic load was ≥10^5^ CFU/mL in all groups. At the end of the incubation period, the planktonic load was ≥10^5^ CFU/mL for the control group. Conversely, 10^3^ CFU/mL were found in group 1 and group 2. A significant reduction (*p* < 0.001) in the planktonic load with respect to control was found in group 3, showing <10^2^ CFU/mL. Similarly, we found differences in the amount of sessile bacteria retrieved from the biofilm formed on catheter surface. Results of the sessile bacterial load at the end of the incubation period are summarized in Table 2. From the surface of catheter segments we retrieved a median (interquartile range) number of CFU/cm of 1.67 × 10^6^ (1.33 × 10^6^) and 1.49 × 10^6^ (0.73) in groups 1 and 2, respectively. On the other hand, in group 3, we note a statistically significant reduction (*p* = 0.004) in sessile bacterial load between group 3 (0.12 × 10^6^ CFU/cm) and control group.

## 4. Discussion

### 4.1. Main Findings

The majority of UTIs are associated with the presence of indwelling urinary catheters (CAUTIs) and are related with the presence of microbial biofilm colonization [6, 18]. The reduction of microbial biofilm development on urinary catheter could be the key in order to avoid CAUTIs. Several clinical strategies have been proposed and tested to prevent biofilm formation, with divergent results. Moreover, the use of antibiotic in prophylaxis or in therapy after catheter substitution is a dangerous practice that is paralleled by a growing frequency of multidrug resistant pathogenic strains [19, 20]. Our preliminary findings show that the use of mother tincture of umbelliferone isolated from* Herniaria hirsuta* 300 mg, arbutin 60 mg extracts, and N-acetylcysteine 150 mg resulted in a significant reduction of microbial colonization and biofilm development on the surface of urinary catheter. This preliminary data, if supported from other* in vitro* and* in vivo* tests, could address the development of new strategies for the prevention of biofilm formation on urinary catheters, thus contributing to the management and reduction of CAUTIs.

### 4.2. Results in Context of Previous Works

The latest Cochrane Review evaluated the influence of the type of indwelling catheter on the development of urinary tract infection in 12,422 hospitalised adults in 25 parallel group trials and 27,878 adults in one large cluster-randomised crossover trial who undergo short-term urinary catheterization [21]. The authors reported that silver alloy-coated catheters were not associated with a statistically significant decrease in symptomatic CAUTI [21]. In particular, authors highlight that nitrofurazone-impregnated catheters reduced the risk of symptomatic CAUTI and bacteriuria, although the magnitude of this reduction was low and hence may not be clinically relevant [21]. Moreover, these catheters were more expensive than standard catheters [21]. In this sense the use of silver alloy-coated catheters or antibiotic-impregnated catheters does not represent the optimal solution for the CAUTI prevention. Recently, Cai et al. documented the efficacy of preparation with* Solidago*,* Orthosiphon*, birch, and cranberry extracts to decrease the incidence of positive urine cultures and a lower amount of biofilm on catheter lumen surface in patients with indwelling urinary catheter [4]. They highlight that the effectiveness of this treatment is due to the specific characteristics of each component, suggesting that the combination of these components can amplify the effect of each one [4]. Several authors demonstrated the* in vitro* efficacy of arbutin and its metabolite hydroquinone against a variety of bacterial strains [11, 22, 23]. Moreover, the efficacy of an oral administration of a single dose of arbutin to inhibit the bacterial growth in urine has been described for the first time in 1970 [24]. Furthermore, Schindler et al. demonstrated that, after oral administration, arbutin is excreted in the urine at the site of action as hydroquinone glucuronide, hydroquinone sulfate, and hydroquinone [11]. In particular, the availability in urine is about 65% of that dose administered [11]. This aspect is very important in order to plan an* in vivo* study for testing the efficacy of this compound in patients with indwelling catheter. The other compounds (N-acetylcysteine and mother tincture of umbelliferone isolated from* Herniaria hirsuta*) are not yet tested in patients with urinary tract infections but they have specific characteristics that can be useful for the bacterial biofilm prevention. N-Acetylcysteine has been purposed as a mucolytic agent, due to the fact that it is able to dissociate mucin disulphide bonds and other disulphide bond cross-linked gel components to reduce viscosity [25]. El-Feky et al. evaluated the effect of ciprofloxacin and N-acetylcysteine alone and in combination against biofilm production and/or preformed mature biofilms on ureteral stent surfaces [26]. Using a standard microbiological analysis and scanning electron microscopy analysis, these authors demonstrated that ciprofloxacin/N-acetylcysteine combination showed the highest inhibitory effect on biofilm production and the highest ability to eradicate preformed mature biofilms [26]. Previously several studies showed that umbelliferone exerts anti-inflammatory, antioxidant, antidiabetic, antinociceptive, and anticancer effects [27]. Moreover, the effects of umbelliferone on bacteria have been also reported [28–30]. Stefanova et al. in an experimental study, using* Salmonella enterica* serovar Typhimurium infection in mice, documented that 7-hydroxycoumarin pretreatment is able to reduce both tissue damaging and immunosuppressive effects of the oxidative stress related to the infection [28]. Lee and coworkers showed that umbelliferone is able to inhibit biofilm* E. coli* O157:H7 formation by more than 80% [29]. Here, we demonstrated for the first time that umbelliferone at the high dose (300 mg) is able to reduce the bacterial growth and biofilm production probably through its ability to induce both biofilm “core” enucleation and diuresis.

### 4.3. Strengths and Limitations of the Study

The present preliminary study shows few limitations to take into account. The first limitation is the limited number of enrolled catheters, due to the study's exploratory design. However, the standardized use of specific bacterial strain and catheter allowed reproducible results. The second limitation is the difficulty of evaluating the efficacy of each compound in decreasing biofilm formation. However, given the fact that bacterial resistance to antibiotics has been increasing, we would like to stress the fact that there is a growing necessity to develop new treatments to reduce catheter bacterial colonization [30].

## 5. Conclusions

The use of mother tincture of umbelliferone 300 mg, extracted from* Herniaria hirsuta*, arbutin 60 mg, and N-acetylcysteine 150 mg is able to reduce* E. faecalis* colonization and biofilm development on the surface of urinary catheter. These preliminary findings can be the basis for clinical studies in patients with indwelling catheter to reduce incidence of UTIs, to reduce the costs for healthcare structures, and to improve the safety of treatment reducing the development of side effects and drug-drug interactions, particularly in elderly patients.

## Figures and Tables

**Table 1:** Group characteristics and results of the quantification of planktonic load in the incubation medium at the beginning and at the end of the experiment.

	Group			
	1	2	3	Control
Compounds in the inoculation broth	Umbelliferone 150 mg	Umbelliferone 150 mg	Umbelliferone 300 mg	5 mL 0.9% sterile saline
	Arbutin 60 mg	Arbutin 60 mg	Arbutin 60 mg	
	N-Acetylcysteine 150 mg	N-Acetylcysteine 400 mg	N-Acetylcysteine 150 mg	

Number of catheters tested	5	5	5	5

Inoculated strain	*E. faecalis*	*E. faecalis*	*E. faecalis*	*E. faecalis*

Planktonic load at the start of experiment (CFU/mL)	≥10^5^	≥10^5^	≥10^5^	≥10^5^

Planktonic load at the end of experiment (CFU/mL)	>10^3^	>10^3^	<100	≥10^5^

Difference with respect to control (*p*)	0.35	0.35	<0.001	1

**Table 2:** Quantification of the sessile bacteria on catheter surface at the end of the experiment.

	Group				
	1	2	3	Control	*p*
CFU/1 cm segment					
Median	1.67 × 10^6^	1.77 × 10^6^	0.12 × 10^6^	3.69 × 10^6^	*0.004*
IQRs	1.33 × 10^6^	0.73 × 10^6^	0.20 × 10^6^	2.28 × 10^6^	
Range (×10^6^)	1.24–2.78	1.44–2.19	0.10–0.44	3.33–5.78	

## References

[1] Bouza E., San Juan R., Muñoz P., Voss A., Kluytmans J. (2001). A European perspective on nosocomial urinary tract infections II. Report on incidence, clinical characteristics and outcome (ESGNI-004 study). *Clinical Microbiology and Infection*.

[2] Hachem R., Reitzel R., Borne A. (2009). Novel antiseptic urinary catheters for prevention of urinary tract infections: correlation of in vivo and in vitro test results. *Antimicrobial Agents and Chemotherapy*.

[3] Feneley R. C. L., Kunin C. M., Stickler D. J. (2012). An indwelling urinary catheter for the 21st century. *BJU International*.

[4] Cai T., Caola I., Tessarolo F. (2014). Solidago, orthosiphon, birch and cranberry extracts can decrease microbial colonization and biofilm development in indwelling urinary catheter: a microbiologic and ultrastructural pilot study. *World Journal of Urology*.

[5] Vergidis P., Patel R. (2012). Novel approaches to the diagnosis, prevention, and treatment of medical device-associated infections. *Infectious Disease Clinics of North America*.

[6] Tenke P., Köves B., Nagy K. (2012). Update on biofilm infections in the urinary tract. *World Journal of Urology*.

[7] Guay D. R. P. (2009). Cranberry and urinary tract infections. *Drugs*.

[8] Mathers M. J., von Rundstedt F., Brandt A. S., König M., Lazica D. A., Roth S. (2009). Myth or truth. Cranberry juice for prophylaxis and treatment of recurrent urinary tract infection. *Urologe A*.

[9] Bellakhdar J., Claisse R., Fleurentin J., Younos C. (1991). Repertory of standard herbal drugs in the Moroccan pharmacopoeia. *Journal of Ethnopharmacology*.

[10] Atmani F., Khan S. R. (2000). Effects of an extract from Herniaria hirsuta on calcium oxalate crystallization in vitro. *BJU International*.

[11] Schindler G., Patzak U., Brinkhaus B. (2002). Urinary excretion and metabolism of arbutin after oral administration of Arctostaphylos uvae ursi extract as film-coated tablets and aqueous solution in healthy humans. *Journal of Clinical Pharmacology*.

[12] Dinicola S., De Grazia S., Carlomagno G., Pintucci J. P. (2014). N-acetylcysteine as powerful molecule to destroy bacterial biofilms. A systematic review. *European Review for Medical and Pharmacological Sciences*.

[13] Kuhn D. M., Chandra J., Mukherjee P. K., Ghannoum M. A. (2002). Comparison of biofilms formed by *Candida albicans* and *Candida parapsilosis* on bioprosthetic surfaces. *Infection and Immunity*.

[14] Mazzoli S. (2010). Biofilms in chronic bacterial prostatitis (NIH-II) and in prostatic calcifications. *FEMS Immunology and Medical Microbiology*.

[15] CLSI (2006). Performance standards for antimicrobial susceptibility testing. *16th Informational Supplement*.

[16] Guiton P. S., Hannan T. J., Ford B., Caparon M. G., Hultgren S. J. (2013). *Enterococcus faecalis* overcomes foreign body-mediated inflammation to establish urinary tract infections. *Infection and Immunity*.

[17] Christensen G. D., Simpson W. A., Younger J. J. (1985). Adherence of coagulase-negative staphylococci to plastic tissue culture plates: a quantitative model for the adherence of staphylococci to medical devices. *Journal of Clinical Microbiology*.

[18] Morris N. S., Stickler D. J., McLean R. J. C. (1999). The development of bacterial biofilms on indwelling urethral catheters. *World Journal of Urology*.

[19] Jarvis W. R. (1996). Preventing the emergence of multidrug-resistant microorganisms through antimicrobial use controls: the complexity of the problem. *Infection Control and Hospital Epidemiology*.

[20] Cai T., Verze P., Brugnolli A. (2016). Adherence to European Association of Urology guidelines on prophylactic antibiotics: an important step in antimicrobial stewardship. *European Urology*.

[21] Lam T. B. L., Omar M. I. M., Fisher E., Gillies K., MacLennan S. (2014). Types of indwelling urethral catheters for short-term catheterisation in hospitalised adults. *The Cochrane Database of Systematic Reviews*.

[22] Ng T. B., Ling J. M. L., Wang Z.-T., Cai J. N., Xu G. J. (1996). Examination of coumarins, flavonoids and polysaccharopeptide for antibacterial activity. *General Pharmacology*.

[23] Robertson J. A., Howard L. A. (1987). Effect of carbohydrates on growth of Ureaplasma urealyticum and Mycoplasma hominis. *Journal of Clinical Microbiology*.

[24] Frohne D. (1970). Untersuchungen zur Frage der harndesinfizierenden Wirkungen von Bärentraubenblatt-Extrakten. *Planta Medica*.

[25] Balsamo R., Lanata L., Egan C. G. (2010). Mucoactive drugs. *European Respiratory Review*.

[26] El-Feky M. A., El-Rehewy M. S., Hassan M. A., Abolella H. A., Abd El-Baky R. M., Gad G. F. (2009). Effect of ciprofloxacin and N-acetylcysteine on bacterial adherence and biofilm formation on ureteral stent surfaces. *Polish Journal of Microbiology*.

[27] Sim M.-O., Lee H.-I., Ham J. R., Seo K.-I., Kim M.-J., Lee M.-K. (2015). Anti-inflammatory and antioxidant effects of umbelliferone in chronic alcohol-fed rats. *Nutrition Research and Practice*.

[28] Stefanova T., Nikolova N., Neychev H., Zlabinger G. (2012). Phagocytosis and killing of Salmonella by 7-hydroxycoumarin activated macrophages. *Immunological Investigations*.

[29] Lee J.-H., Kim Y.-G., Cho H. S., Ryu S. Y., Cho M. H., Lee J. (2014). Coumarins reduce biofilm formation and the virulence of *Escherichia coli* O157:H7. *Phytomedicine*.

[30] Avorn J., Monane M., Gurwitz J. H., Glynn R. J., Choodnovskiy I., Lipsitz L. A. (1994). Reduction of bacteriuria and pyuria afteringestion of cranberry juice. *The Journal of the American Medical Association*.

